# RheoScale 2.0: Revealing the hidden roles of protein positions via substitution patterns

**DOI:** 10.1002/pro.70804

**Published:** 2026-09-21

**Authors:** David H. Liu, Shwetha Sreenivasan, Carter J. Gray, Hannah C. Cleveland, Liskin Swint‐Kruse

**Affiliations:** ^1^ Department of Biochemistry and Molecular Biology University of Kansas Medical Center Kansas City Kansas USA; ^2^ Department of Electrical Engineering and Computer Science University of Kansas‐Lawrence Lawrence Kansas USA; ^3^ Present address: Cellular Engineering Group National Institute of Standards and Technology Gaithersburg Maryland USA; ^4^ Present address: Medical Scientist Training Program, Carver College of Medicine University of Iowa Iowa City Iowa USA

**Keywords:** high‐throughput datasets, protein engineering, rheostats, sequence‐structure–function relationships

## Abstract

A central challenge in molecular biology is understanding how amino acid substitutions modulate various features of protein function and stability. To illuminate the complexities of this relationship, high‐throughput (HTP) assays are increasingly used to assess site‐saturating mutagenesis libraries. A common downstream analysis is to average the set of 20 outcomes at each amino acid position for comparison with structural and evolutionary features. Average values clearly identify positions that tolerate most substitutions (neutral positions) and positions where most substitutions abolish activity (toggle positions). However, average values conceal the existence of rheostat positions, where different amino acid substitutions sample a wide range of outcomes. To quantitatively identify rheostat positions, we previously developed a histogram‐based analysis that we here expand by: (i) incorporating new position classes observed in experimental studies of rheostat positions; (ii) formalizing a hierarchy of class assignments; (iii) refining error‐based identification of neutral positions; and (iv) statistically assessing the robustness of class assignments to changes in experimental and computational parameters. RheoScale 2.0 is implemented in Excel and newly implemented in Python for facile integration with existing HTP pipelines; all parameters are customizable. Example analyses are shown for three HTP datasets of the SARS‐CoV‐2 papain‐like protease. Results illustrate two aspects that influence interpretation of HTP data: First, position assignments (and substitution outcomes) depend highly on the measured feature. Second, many protein positions play multiple roles in the sequence‐structure–function relationship. The recognition of varied position roles will advance understanding of pathogen evolution, protein engineering, and variant interpretation for personalized medicine.

## 1 INTRODUCTION

Advances in personalized medicine and protein engineering, as well as recognizing which changes are significant for pathogen evolution, require understanding how variations in amino acid sequence impacts protein function and/or stability. Despite decades of effort, outcomes from single substitutions cannot yet be reliably predicted by computational algorithms (e.g., Turina et al. (2025), Zhang et al. (2024), Livesey and Marsh (2025), López et al. (2025), Miller et al. (2017)). This challenge is, in large part, due to difficulties identifying biologically‐meaningful substitutions at evolutionarily non‐conserved positions and/or positions far from binding sites (e.g., Sreenivasan et al. (2025), Swint‐Kruse and Fenton (2024), Swint‐Kruse et al. (2021), Tang and Fenton (2017)). Historically, these types of positions were overlooked in substitution experiments and, thus, under‐represented in early computational training/test sets. To address this gap, over the past decade, numerous high‐throughput (HTP) approaches have been developed to generate whole‐protein measurements of substitution effects (e.g., Flynn et al. (2024), Amorosi et al. (2021), Wu et al. (2024), Tack et al. (2021), Wu et al. (2025)).

**FIGURE 1:**
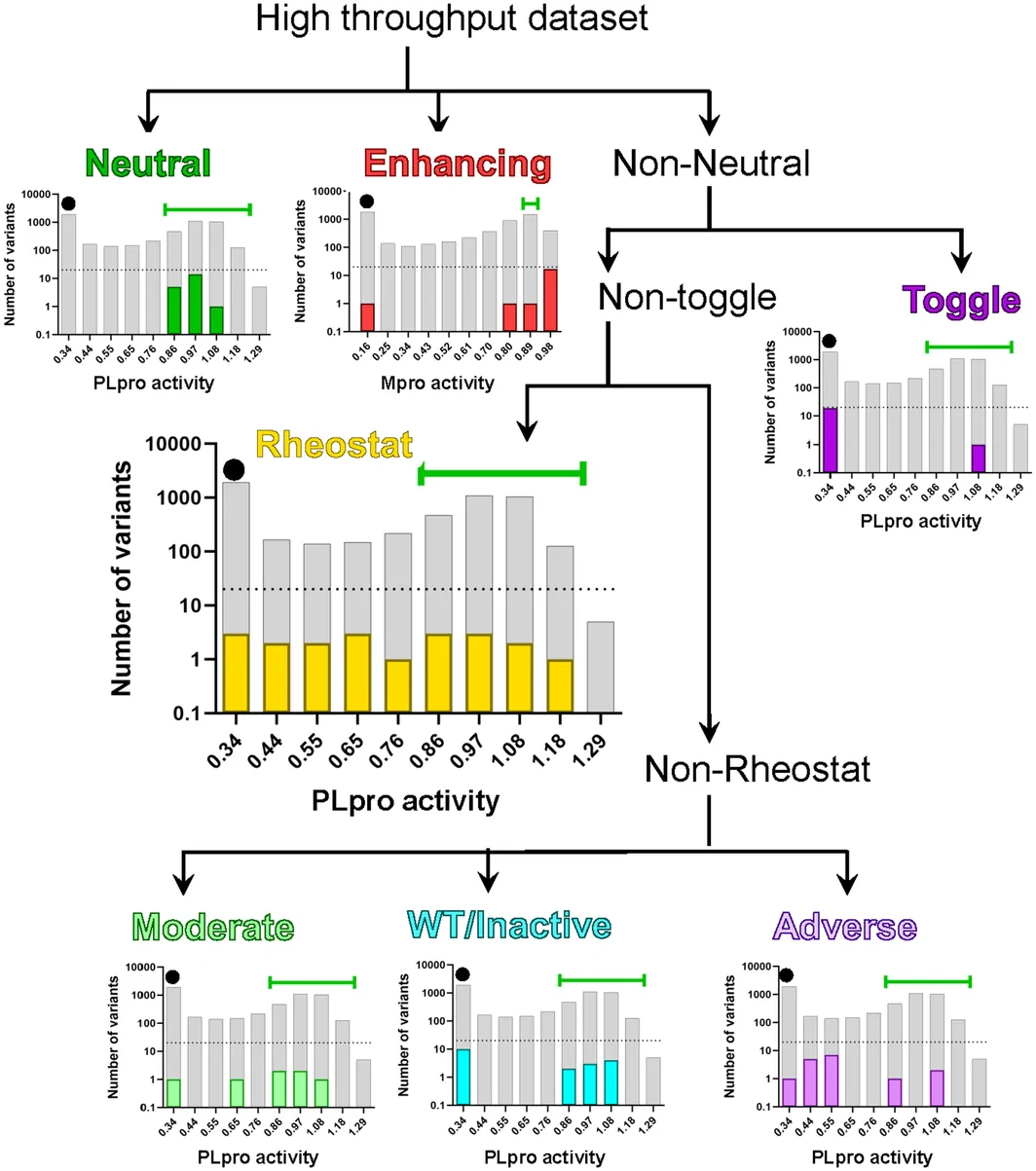
Hierarchical assignment of protein positions using RheoScale 2.0. HTP datasets are frequently presented as histograms. The gray histograms shown here represent the outcomes for all possible substitutions in SARS‐CoV2 PLpro (Wu et al., 2024) (or its Main protease, Mpro (Flynn et al., 2024)). RheoScale 2.0 uses these histograms to assess the overall substitution sensitivities of individual positions. Analyses use three reference points: (i) the range of values observed for wild‐type replicates (WT; green horizontal bars), (ii) the value corresponding to zero activity (black dot on the left‐most bar), and (iii) a value for the “most active” end of the range, which is usually determined from empirical observations. Activity values on the *x*‐axes correspond to the upper limits of each histogram bin, in the logarithmic units reported by the studies' authors. The *y*‐values are the number of variants observed and are also shown on log scale. Each position's assignment is based on the pattern observed for its set of substitution outcomes. Assignments are made hierarchically as shown. Examples for each assignment are: Neutral (PLpro position 2); Enhancing (SARS‐CoV‐2 Mpro position 151); Toggle (PLpro position 109); Rheostat (PLpro position 82); Moderate (PLpro position 31), WT/Inactive (PLpro position 139), and Adverse (PLpro position 10). Parameters used to classify PLpro positions are provided in the example analysis files posted at github https://github.com/liskinsk/RheoScale‐calculator; RheoScale analysis of Mpro was reported by Sreenivasan et al (Sreenivasan et al., 2025).

These HTP approaches yield a wealth of data but also raise analytical challenges: how can outcomes for multiple substitutions per position be meaningfully compared to structural features or evolutionary patterns? For these comparisons, a position‐based analysis is required. One common approach uses each position's set of substitution outcomes to calculate the average value. This metric effectively discriminates “toggle” positions, where most substitutions severely impair one or more protein features (e.g., binding affinity, allosteric regulation), from “neutral” positions, where substitutions have minimal effects (Figure 1). However, average values might fail to discriminate “rheostat” positions (Figure 1, yellow histogram), where substitutions sample a wide range of outcomes. This oversight leads to missed opportunities for understanding the protein sequence‐structure–function relationship.

Indeed, accumulating experimental evidence shows that rheostat positions are widespread (Swint‐Kruse & Fenton, 2024). They occur in many types of proteins, including globular and intrinsically disordered transcription factors, globular enzymes, integral membrane transport proteins, and extracellular receptors. In several proteins, >30% of their positions exhibit rheostatic behavior (Sreenivasan et al., 2025; Swint‐Kruse et al., 2021; Swint‐Kruse et al., 2023). This observation is mirrored in analyses of proteins in the Swiss‐Prot and EXPV datasets, where >30% of amino acid positions were predicted to be rheostat positions by the “funtrp” algorithm (Miller et al., 2019).

Substitutions at rheostat positions can also modulate a wide variety of protein features, including: ligand binding, allosteric regulation, substrate transport, ligand specificity, and protein stability (Swint‐Kruse & Fenton, 2024). Some rheostat positions simultaneously modulate multiple protein features (Swint‐Kruse & Fenton, 2024). Substitution outcomes on function seldom correlate with amino acid physicochemical properties and show no consistent relationships to binding sites or solvent exposure (Swint‐Kruse & Fenton, 2024). Instead, the structural regions around rheostat positions appear to be “plastic,” accommodating substitutions via localized changes (Swint‐Kruse & Fenton, 2024). Other evidence suggests that substitutions at rheostat positions alter protein dynamics (Campitelli et al., 2021; Campitelli et al., 2024; Kariyawasam et al., 2025).

Finally, many computational algorithms misclassify substitution outcomes at rheostat positions (Miller et al., 2017). This surely leads to missed and misdiagnoses in personalized medicine and impedes our understanding of pathogen evolution. A better understanding of rheostat positions would also advance protein engineering: (i) Rheostat positions provide ideal targets for fine‐tuning various protein features, such as activity, ligand specificity, and/or stability and (ii) substitutions at rheostat positions often exhibit gain‐of‐function outcomes (Swint‐Kruse & Fenton, 2024). For example, changes at rheostat positions were critical for optimizing the functions of novel chimeric transcription factors (Gray et al., 2026; Meinhardt et al., 2013), and a gain‐of‐function substitution at a rheostat position in a bile acid transport protein is a medically‐relevant polymorphism (Ruggiero et al., 2021).

Clearly, it is vital to objectively identify rheostat positions in experimental datasets for further study. To that end, we previously developed a histogram‐based analysis to quantitatively distinguish rheostat, neutral, and toggle positions (Hodges et al., 2018). Since then, we have identified new position classes (Page et al., 2022; Sreenivasan et al., 2025; Swint‐Kruse et al., 2021), formalized a hierarchy for assigning position types, and improved error‐based identification of neutral positions (Martin et al., 2020). Here, we unify these advances into RheoScale 2.0 and statistically assess effects of experimental and computational parameters on position assignments. These new features are integrated into the Excel version of the calculator and newly implemented as a Python package for facile integration into HTP pipelines.

## 2 METHODS

### 2.1 Calculator overview

For a protein's set of single amino acid substitutions, RheoScale 2.0 uses a modified histogram‐analysis of assay values to classify the roles of individual protein positions (Figure 1). To that end, the histogram for each position is used to calculate four distinct scores—neutral, rheostat, toggle, and enhancing; mathematical definitions for these scores are included in the instruction sheets that accompany the calculators and in (Hodges et al., 2018; Martin et al., 2020; Miller et al., 2017; Sreenivasan et al., 2025; Swint‐Kruse et al., 2021). Each set of scores is then used to classify each position using the hierarchy described below. For these assignments, default score thresholds are based on prior experimental studies; for bespoke analyses, override features are available for all parameters, as discussed further below.

RheoScale 2.0 is available on GitHub in two formats:

1. An interactive Excel workbook. A “small” version of the workbook analyzes up to 19 substitutions for up to 20 unique positions plus wild‐type (WT), returning scores, assignments, and histograms for all. The “large” version of the workbook returns scores and assignments for the smaller of either all substitutions at 350 positions or 6000 substitutions. The first worksheet of each workbook contains detailed instructions on its use.
2. A Python package. The “rheoscale” python package generates scores, histograms, and assignments for large datasets. It can be integrated into pipelines for analyzing HTP data, supports integration with Jupyter notebooks, and is accompanied by a readme file that describes its implementation. This program has three dependencies: numpy (Harris et al., 2020) and pandas (McKinney, 2010), and matplotlib (Hunter, 2007). The Python implementation of RheoScale 2.0 is available through PyPI and can be installed using Python's package manager, pip (https://pypi.org/project/rheoscale/). Unlike the Excel workbooks, the Python script has no limit on the number of substitutions and positions that can be analyzed; its runtime and maximum dataset size will depend on available computational resources.

Both Excel and Python implementations can be found at https://github.com/liskinsk/RheoScale-calculator.

### 2.2 Definitions and hierarchy of position classes

1. In RheoScale 2.0, the first decision is to determine whether a position is neutral for the measured feature. The default definition is that ≥70% of these positions' substitutions have WT‐like outcomes, using the “neutral bin” described in the next section. The rationale for this default threshold was extensively discussed in Martin et al (Martin et al., 2020). As further described below, the default threshold can be changed by the user when deemed appropriate.
2. Next, the neutral bin is used to assess how many substitutions are “better” than WT. If ≥80% of a position's substitutions have values “better” than the neutral range, this position is defined as enhancing (Sreenivasan et al., 2025). To date, we have only encountered a few enhancing positions and the biological implications of their existence are not yet clear. One possibility is that a trade‐off between two protein features constrained the evolution of the natural protein. For example, a position classified as enhancing in a stability assay may be a toggle position for activity and, consequently, would be selected against during evolution. Alternatively, the WT protein could also be “caught” at an unusual evolutionary step, after a recent change created new enhancing possibilities that have not yet been explored.

The remaining non‐neutral positions are further classified according to the following logic:

3 If most of a position's substitutions lack detectable signals, it is designated as a toggle position. The default threshold in the calculator, 64%, was derived from a database for *Escherichia coli* lactose repressor protein (Markiewicz et al., 1994; Miller et al., 2017). This suggested threshold was chosen to allow physiochemically “similar” amino acids to substitute for each other, as has been experimentally observed for positions that are intolerant to most substitutions. Again, the default threshold can be changed by the user when deemed appropriate.

Our choice to assign toggle positions before rheostat positions meets the logical requirement of assigning the extreme outcomes (toggle and neutral positions) to provide the context needed for assigning positions with the intermediate outcomes (e.g., rheostat positions). As such, in the hierarchy of position assignments, toggle assignments take precedence even if the remaining substitutions surpass the rheostat score threshold described below.

4 The set of substitutions for a rheostat position widely samples the range of possible values for the measured feature. This is quantified via the rheostat score, which is calculated by scoring occupancy of each histogram bin as empty (0) or filled (1), regardless of how many substitutions occupy the bin. Simplistically, the set of substitutions at rheostat positions sample at least half of the possible range (Hodges et al., 2018); the use of weighted rheostat scores privileges substitutions that differ most from both the WT and inactive variants (Hodges et al., 2018). Weighted rheostat scores (described in Hodges et al. (2018). and the instructions that accompany the RheoScale 2.0 calculators) are used in the assignment hierarchy with a default threshold of 0.5.

In addition to the three original assignments defined using the thresholds described above (rheostat, toggle, and neutral), two intermediate behaviors have been observed in experimental datasets:

5 At a moderate rheostat position, its set of substitutions has non‐neutral outcomes, but (i) sample less than half of the possible range and (ii) the average of the experimental values is closer to the WT than to inactive variants (Swint‐Kruse et al., 2021).
6 Adverse positions, like moderate positions, have substitutions with non‐neutral outcomes that sample less than half of the possible range. However, the average outcome for their sets of substitutions is closer to the inactive value than to WT (Sreenivasan et al., 2025).

The existence of these six position classes indicates that, in addition to the continuum of outcomes available from single substitutions at rheostat positions, the positions in a protein can fall along a continuum of position classes. Efforts are underway to discriminate the biophysical bases of the different position types (e.g., Campitelli et al. (2024), Fenton et al. (2022), Kariyawasam et al. (2025)). In future studies, it will be particularly interesting to determine whether the biophysical characteristics of moderate positions fall on the continuum between neutral and rheostat positions and whether those of adverse positions are between rheostat and toggle positions.

7 Finally, some positions have shown a “WT/Inactive split” behavior. That is, around half of their substitutions are like WT and the other half lack detectable signal; very few show intermediate outcomes (Page et al., 2022). This assignment may be a hallmark of altered protein stability (O'Neil et al., 2024)

### 2.3 Key analysis parameters

For RheoScale 2.0 analyses, the user must balance considerations arising from: the numbers of variants available for each position, the error of the experimental data, the width of the neutral bin, the total range of the histogram, the number of bins used to calculate the rheostat score, and the score thresholds used to make assignments. Although the calculator uses the input data to recommend default values as reasonable starting places for various parameters, these parameters should be systematically varied for each dataset to assess the robustness of position assignments. Edge cases—which have been rare in our prior studies—require user interpretation.

Detailed descriptions of the effects of experimental error on the calculations and position assignments are described in Hodges et al. (2018), Martin et al. (2020). Additional discussions of the other parameters are in the next paragraphs.

1. To identify rheostat positions, experimental data should contain multiple variants for each amino acid position analyzed. Although 19 substitutions (plus WT) per position are ideal, we previously reasoned that 10 substitutions per position should be sufficient to estimate the locations of rheostat and toggle positions (Hodges et al., 2018). We also identified rheostat positions with as few as 5 variants, reasoning that since the available set sampled more than half the histogram range, having more variants would not change the rheostat assignment (Campitelli et al., 2024). Unambiguous assignment of neutral positions requires many variants per position and assays of multiple protein features; it is hard to prove a negative (Martin et al., 2020)

**FIGURE 2:**
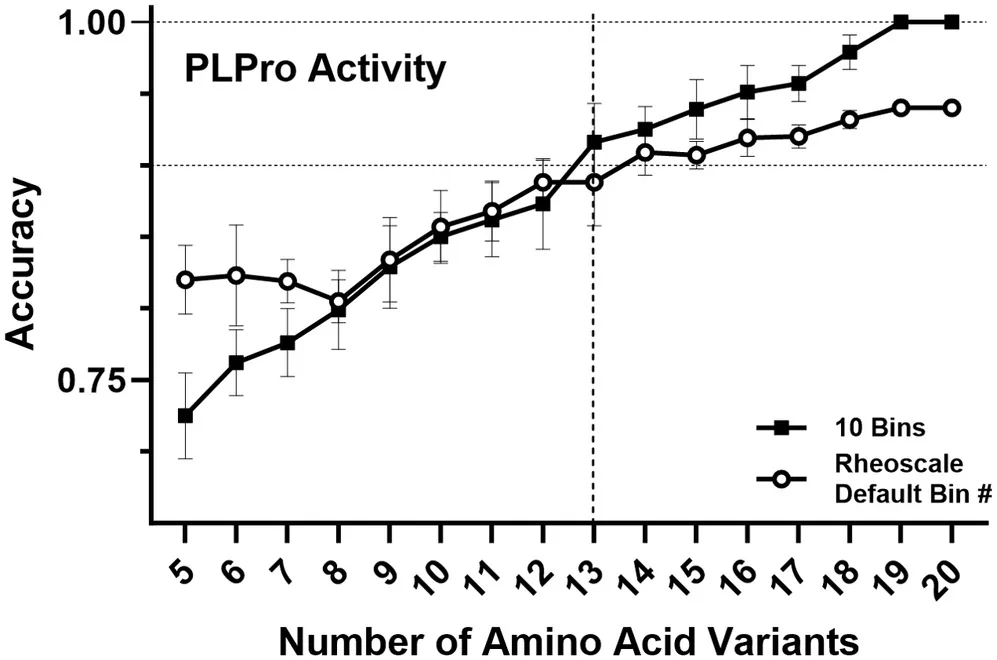
RheoScale 2.0 assignments are robust to changing variant numbers. To assess how the number of available variants alters position assignments, we used positions in the HTP dataset of PLpro activity for which all 20 variants are available. The variant set for each position was randomly subsampled to generate 10 replicates for each of 5–20 variants. RheoScale analyses were carried out for each subsample using either 10 bins (squares) or the default number of bins recommended by RheoScale (open circles); note that the recommended default bin number is based on the number of available variants, as described in Hodges et al. (2018), and thus differs with variant number. For each variant number, assignment accuracy was compared to the assignments using all 20 variants and 10 bins as “truth,” and the average and standard deviation for each set of subsamples is plotted. All class assignments used default score thresholds. Dashed lines are to aid visual inspection of the data.

To further assess the relationship between the number of available variants and the robustness of RheoScale 2.0's assignments, we here used random sampling of the PLpro HTP activity dataset (Wu et al., 2024). The PLpro library contains variants for 315 positions. Of these, 96 positions have assay results for all 20 variants (19 substitutions plus WT), 190 positions have results for 15–19 substitutions, 29 have results for 10–14 substitutions, and 2 positions have results for 7 substitutions. For this exercise, we selected the positions with all 20 substitutions and used random sampling to generate 15 subsets with 5–19 substitutions per position. For each subset, random sampling was repeated 10 times, and two RheoScale analyses were carried out: (i) using 10 histogram bins (Figure 2, filled squares) and (ii) using the default bin number recommended by Rheoscale 2.0 (Figure 2, black, open circles). The assignment accuracies were then calculated relative to assignments using all 20 variants for each position.

For each set of 10 random replicate subsets, we then calculated average accuracies and standard deviations (Figure 2; https://github.com/liskinsk/RheoScale-calculator/tree/main/Plpro%20HTP%20examples). As expected, assignment accuracy increased with the number of available variants. Nevertheless, when 10 histogram bins were used, assignments approached 90% accuracy with only 13 variants per position and >70% of the changed assignments were to an adjacent category (e.g., a rheostat assignment changed to either an adverse or a moderate assignment) for all sample sizes. Using the RheoScale 2.0 default‐recommended bin numbers, the missed assignments included more non‐adjacent categories; however, there were fewer incorrect assignments overall. Even 7 variants per position showed >80% accuracy when the RheoScale 2.0's recommended bin number was used (Figure 2).

2 The width of the neutral bin, which is distinct from the histogram bins, is the key parameter for assessing whether variants are equivalent to WT and, in turn, whether a position is neutral (Martin et al., 2020; Wu et al., 2019). The neutral bin is centered on the value measured for the WT protein; the default width of the neutral bin is ±2 standard deviations of the WT error (or four times the error override for the full dataset). If no error is designated, the neutral bin width is set to twice the width of the histogram bin that is used to calculate rheostat and toggle scores. The calculator also provides an opportunity for the user to manually define the neutral bin size. Variants with values that fall in the neutral bin are designated as “WT‐like.”
3 The most important parameter for assigning non‐neutral positions is the histogram range. In RheoScale 2.0, the range defaults to the minimum and maximum of the experimental data. If experimental data do not reflect the actual range, for example, the measured outcomes only sample a portion of the assay's accessible range, the user can enter override values. Various examples that require user‐determined values, including strategies for assigning “dead” values that define the bottom of the range, are in the documentation that accompanies the RheoScale 2.0 calculators.
4 The recommended number of histogram bins is calculated from the average error for the dataset and the number of variants available for each position in the dataset (Hodges et al., 2018). Since rheostat positions are defined by having substitutions with intermediate loss of signal, the resolution of the experimental data is critical. Ideally, at least three histogram bins will contain intermediate values. The user can also choose a bespoke bin number; empirically, 10 histogram bins work well for many HTP datasets.

**FIGURE 3:**
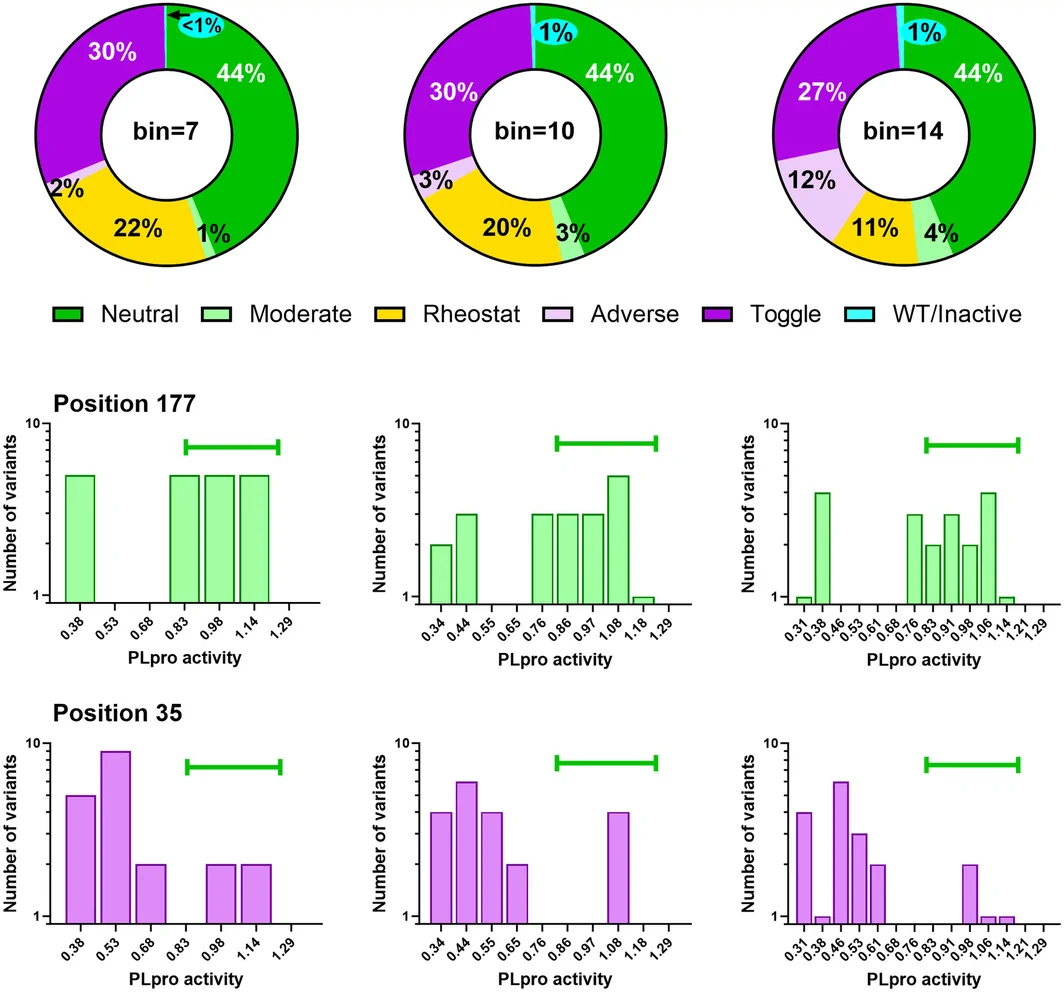
Effects of changing bin numbers on position assignments. RheoScale analyses were carried out for the PLpro activity data (Wu et al., 2024), with bin numbers ranging from 7 to 14. Position classes are colored as follows: Neutral, green; moderate, light green; rheostat, yellow; adverse, light purple; toggle, purple; and WT/inactive split, cyan. Histogram bins are labeled with their upper limit, as in Figure 1. The most frequent assignment changes were when rheostat positions changed to an adjacent category (either adverse or moderate). The histogram plots show examples for this behavior using 7, 10, and 14 bins: Position 177 changes from moderate to rheostat and back to moderate; position 35 changes from rheostat to adverse and back to rheostat. Note that the fraction of neutral scores does not change because these assignments are made using a separate “neutral bin” (green horizontal bar on the histograms).

Indeed, iterating RheoScale analyses with different bin numbers has shown that most position assignments are not very sensitive to this parameter (e.g., Swint‐Kruse et al. (2025)). For a given histogram range, decreasing the bin number increases the bin width. Although this can be appropriate for accommodating larger experimental errors, it is also more likely that a set of substitutions will sample a large fraction of the available bins. These two features usually offset each other, resulting in the same position assignment. This was seen for assignments using activity values for the full set of PLpro positions (with 7–20 variants) and with bin numbers ranging from 7 to 14 (e.g., Figure 3). The exception is sparse datasets (e.g., Figure 2; 5–7 variants per position), where using the smaller, RheoScale‐recommended number of bins has better accuracy than a larger number that forces several bins to be empty (i.e., five variants can never fully sample 10 bins.).

Of the PLpro positions that changed assignment classes in this exercise (51 of 315, which is 16%), the majority changed to an adjacent category (e.g., rheostat to moderate). This is expected when a continuous property is subjected to thresholds (Swint‐Kruse, 2016), which are here imposed by bin boundaries and assignment score thresholds. At the largest bin number used in this exercise (14), most of the reassignments were rheostat positions to moderate or adverse positions. We considered whether the reassigned positions were dominated by positions with <14 variants, but the fraction of changed assignments in this subset (5 of 20, or 25%) was only marginally higher than the overall fraction of changes (16%). This suggests that most reassignments occur when substitution values fall near the boundaries of a bin (which changes with bin number).

For the PLpro activity results presented below, we used 10 bins to balance the competing interests of (i) accommodating experimental error (requiring fewer histogram bins) and (ii) avoiding over‐predicting the number of rheostat positions (via more histogram bins). For other datasets, another strategy would be to vary the bin number and choose the consensus assignment for each position.

5 For sparse or low resolution datasets, assignment score thresholds should also be considered. Stricter thresholds (such as requiring 75% of variants to be inactive when assigning toggle positions) provide more confidence in position assignments, especially for sparse experimental datasets (e.g., Campitelli et al. (2024), Swint‐Kruse et al. (2021)). As with bin numbers, assignments should be iteratively evaluated for their robustness to different thresholds; position assignments that are not robust should be manually inspected.

## 3 RESULTS AND DISCUSSION

To demonstrate the utility of RheoScale 2.0, we used three deep mutational scanning datasets for a site‐saturating mutagenesis library of SARS‐CoV‐2 PLpro (Wu et al., 2024; Wu et al., 2025). (i) The activity assay measured in vivo proteolysis of a fluorescent reporter protein after PLpro expression was induced in mammalian cells (Wu et al., 2024). (ii) Changes in in vivo abundance were measured using a construct that fused PLpro directly to a fluorescent reporter protein (Wu et al., 2024). (iii) Finally, another implementation of the activity assay included enzyme inhibitors; the dataset used here was generated by the original authors by combining HTP assays using two different inhibitors (Wu et al., 2025). Analyses of these datasets reveal the diversity of position assignments, as observed in many other proteins (Swint‐Kruse & Fenton, 2024). All RheoScale 2.0 assignments for PLpro, as well as a table compiling assignments from the three assays, are available at https://github.com/liskinsk/RheoScale-calculator.

**FIGURE 4:**
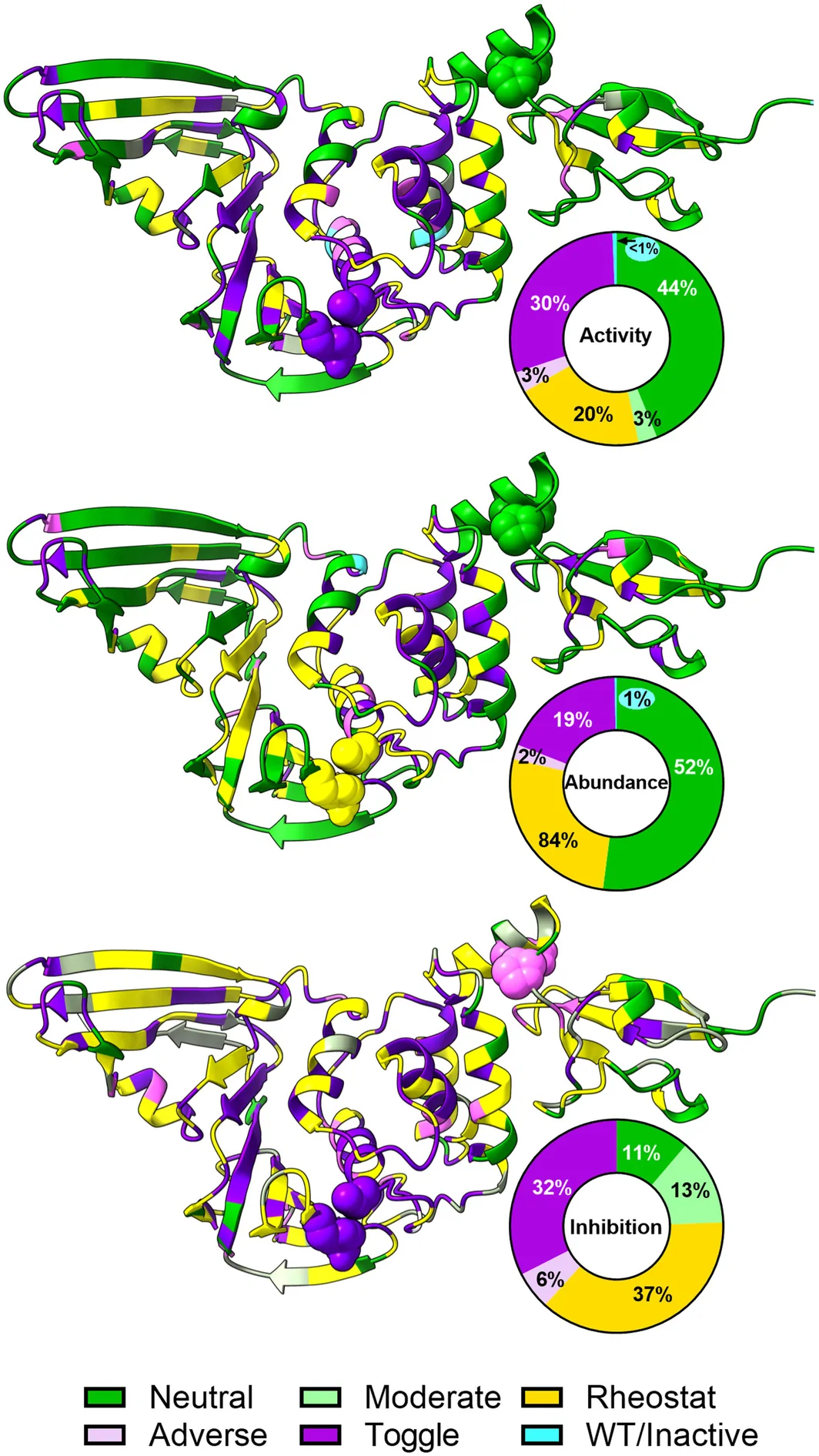
PLpro positions exhibit assay‐dependent assignments. Ribbon representations of SARS‐CoV‐2 PLpro (PDB: 8VEC (Wu et al., 2024)) are color‐coded according to their RheoScale 2.0 position assignments for (top) protease activity, (middle) in vivo abundance, and (bottom) activity in the presence of inhibitors. Donut charts summarize the fraction of PLpro positions assigned to each position class. Position classes are colored as follows: Neutral, green; moderate, light green; rheostat, yellow; adverse, light purple; toggle, purple; and WT/inactive split, cyan. The catalytic triad residues C111, H272, and D286 are shown as purple spheres on the structure showing activity and inhibition data and yellow on the abundance data; these positions are classified as toggle in the activity and drug escape but rheostat in abundance. Positions 59 and 67 are also highlighted as spheres (pink on the structure showing inhibition assignments); these positions are near the domain interface and are neutral in the activity and abundance datasets but become adverse in the inhibitor dataset. Structure images were generated using UCSF ChimeraX (Pettersen et al., 2021).

**FIGURE 5:**
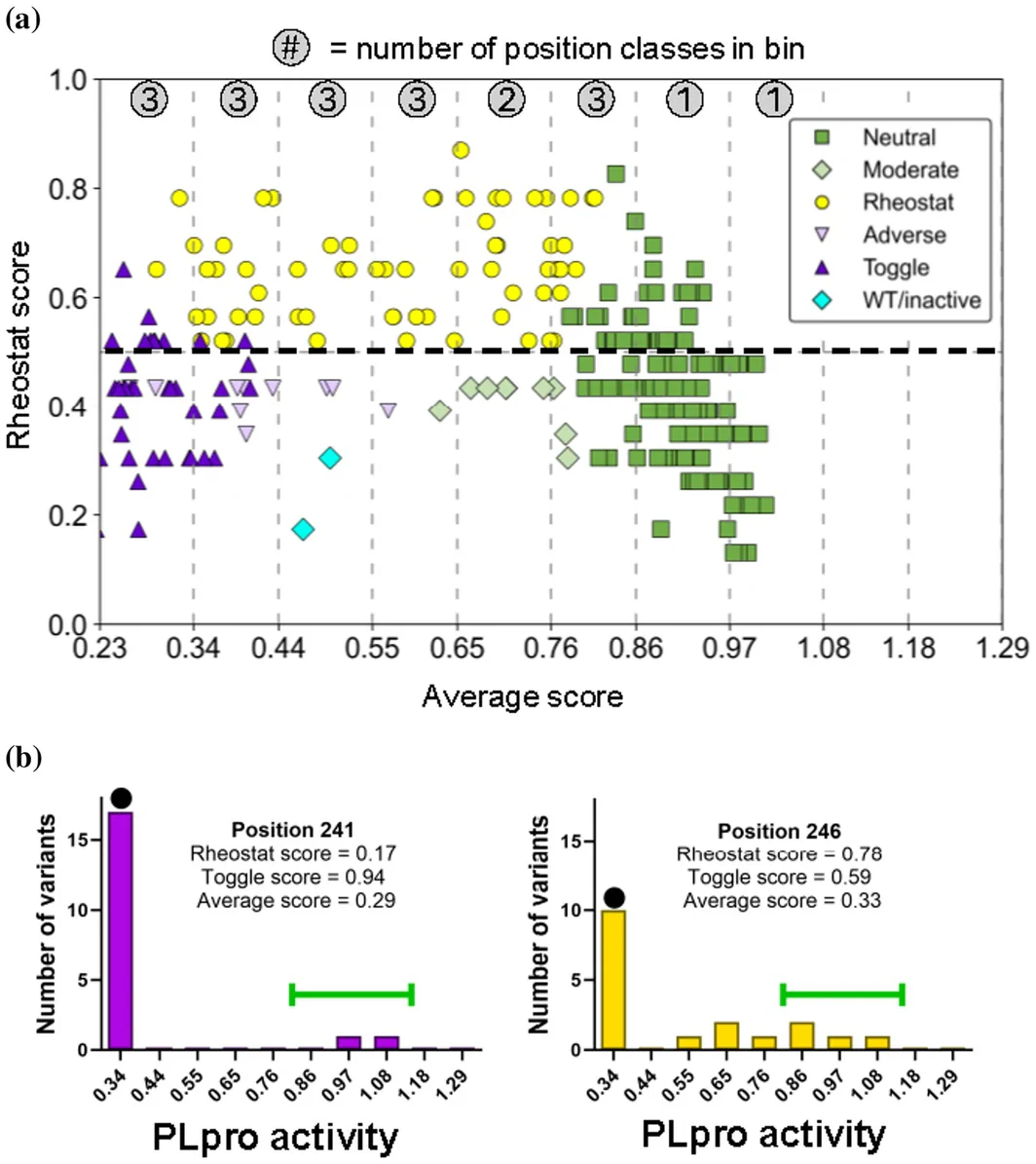
RheoScale 2.0 assignments using SARS‐CoV‐2 PLpro activity data (Wu et al., 2024). (a) Rheostat scores versus average scores for each PLpro position. Each point on the XY plot represents a unique PLpro position; symbols correspond to position assignments. The dashed horizontal line indicates the threshold of 0.5 that is used to assign rheostat positions (after neutral and toggle assignments are made). The dashed vertical lines divide the average values into the same bins as in Figure 1, and the numbers in gray circles denote how many position classes are represented for each bin. An example of two positions with similar averages but different rheostat scores are shown in (b). Position 241 (left) demonstrates toggle behavior; position 246 (right) displays rheostat behavior. Other features of these plots are as described in Figure 1. In (a) and (b), the values on the *x*‐axes correspond to the upper bin limits.

In the PLpro protease activity data (Figure 4), ~20% of positions were rheostat positions. Similar to other proteins (Swint‐Kruse & Fenton, 2024), rheostat positions are dispersed across the structure, which underscores the fact that a position's substitution sensitivity cannot be inferred from its location. As anticipated, RheoScale 2.0 analyses further parsed PLpro positions with similar average scores (e.g., Figure 5) into different position classes. This is important because substitutions at rheostat positions appear to arise from special biophysical characteristics (e.g., Campitelli et al. (2024)).

Comparing results for activity and abundance assays illustrates that each position on a protein's scaffold can play specific roles. For example, PLpro positions that are classified as toggle positions for both activity and abundance may primarily contribute to protein stability. Other positions show no effect on abundance but do show effects on activity, suggesting that these positions contribute primarily to function. Still, other positions affect both features, and as observed for Mpro (Sreenivasan et al., 2025), the altered activity may arise from simultaneously changing both function and abundance. Additional “layers” of position roles might be expected if PLpro were to be assayed for different activities such as de‐ubiquitinylation and de‐ISGylation of host proteins; these functions use the same active site but different substrate binding sites (Klemm et al., 2020). We have observed similarly complex relationships between position roles and distinct functional features in several other proteins (O'Neil et al., 2024; Swint‐Kruse et al., 2021; Swint‐Kruse et al., 2025; Swint‐Kruse & Fenton, 2024; Wu et al., 2019).

The comparison of activity and abundance data also highlights the importance of understanding assays' detection limits. In general, one would expect that a change in activity would be constrained by any change in abundance. That is, substitutions with undetectable abundance should not have activity, and thus positions that are toggle for abundance should not be rheostatic for activity. However, some PLpro positions that are classified as toggle for abundance are rheostat positions for activity. A plausible explanation for this apparent paradox is that the activity assay is more sensitive than the abundance assay. This difference precluded calculations to dissect functional information from the activity score (e.g., Sreenivasan et al. (2024), Sreenivasan et al. (2025), Swint‐Kruse et al. (2025)), which is a composite of both function and abundance.

When results for the inhibitor assay are compared to the activity assay, many PLpro positions show greater sensitivity to substitution and more are classified as rheostat positions (37% vs. 20%; Figure 4). This can be explained by the inhibitor lowering the effective in vivo concentration of active PLpro for the majority of substitutions. This observation also suggests that the first activity assay—with data collected at a single time point 24 h after PLpro induction—was performed under “V_max_” conditions that obscured the effects of many substitutions. Similar findings were observed from comparisons of two Mpro HTP datasets assayed at different induction times (Flynn et al., 2022; Flynn et al., 2024; Sreenivasan et al., 2025).

In addition, one might expect some PLpro substitutions to be more or less sensitive to inhibitors than others. Indeed, Call and colleagues identified five positions with substitutions resistant to inhibitor (Wu et al., 2024); four of these positions (PLpro 164, 167, 208, and 269) are classified as rheostat positions in the inhibitor assay, while the fifth (268) is classified as a moderate position. These five positions were also the locations of resistance mutations for more potent inhibitors (Wu et al., 2025), as were positions 266 and 267, which are also rheostat positions in the weak inhibitor assay; a third position for an escape mutant (301) was a toggle position. Thus, 75% of the positions with escape mutants were rheostat positions, which is essentially double the probability of sampling a rheostat position by random chance (37%; see Figure 3 legend). This suggests that amino acid changes at rheostat positions can play important roles in viral evolution and drug escape, and identification of rheostat positions could be beneficial for focusing further studies.

RheoScale 2.0 analyses also identify two positions with higher‐than average sensitivity to inhibitors (PLpro positions 59 and 67). Intriguingly, these two positions are near each other on the structure and near a domain interface (Figure 4, pink spheres in inhibition data). Biochemical and biophysical studies of these and other rheostat positions should be fruitful for understanding the sequence‐structure–function relationship of this PLpro construct at a molecular level.

Finally, we used the PLpro analyses to consider how different types of experimental data (e.g., Figure 4) correlate with predictions of position assignments. The funtrp algorithm predicts whether positions exhibit neutral, toggle, or rheostat behavior (Miller et al., 2019). This algorithm was trained on HTP datasets for five proteins, each of which measured substitution effects on a feature specific to that protein (or, in cell‐based assays, a combination of features such as function and abundance). Additional input features included (i) a variety of biophysical characteristics about each amino acid and its predicted location in the protein structure, (ii) the number of possible single nucleotide polymorphisms that lead to a missense mutation, and (iii) ConSurf (Ashkenazy et al., 2010, 2016) scores, which are derived from phylogenetic conservation analyses of multiple sequence alignments. ConSurf scores contributed the most to funtrp predictions (Miller et al., 2019). Because of this, we chose to focus our comparisons on the PLpro activity and abundance measurements, reasoning that, since inhibitors were not part of PLpro's natural evolution, they would not leave a “signature” in the multiple sequence alignment of PLpro homologs.

We also considered that, in training funtrp, the thresholds used for classifying neutral and toggle positions differed from the default RheoScale thresholds: Neutral positions were designated when “all but one” variants were wild‐type (Miller et al., 2019). Toggle positions were designated when “all but two” variants were detrimental (Miller et al., 2019). Since the number of variants in the funtrp training dataset ranged from 6 to 20 per position, converting these to fraction‐based thresholds comparable to those of RheoScale 2.0 spans a range: The neutral threshold could range from 0.80 to 0.95 and the toggle threshold could range from 0.67 to 0.9. Since the lower ends of those ranges are similar to RheoScale 2.0 default values, we repeated the comparisons using the stricter thresholds of 0.9 for both neutral and toggle assignments.

Our first question was how funtrp handles positions experimentally assigned to the moderate and adverse classes, since these were not described when funtrp was developed. Using RheoScale's default score thresholds, the experimental abundance data had seven adverse positions; funtrp predictions for these positions were split among rheostat and toggle classes, both of which are reasonable assignments. In the activity data, five of the nine moderate and six of the 10 adverse positions were assigned to the rheostat category, two moderates were predicted to be neutral, and three adverse were predicted to be toggle. Again, these are reasonable, “correct” predictions. However, two positions that were moderate for activity were predicted to be “toggle” and one adverse position for activity was predicted to be “neutral.”

**FIGURE 6:**
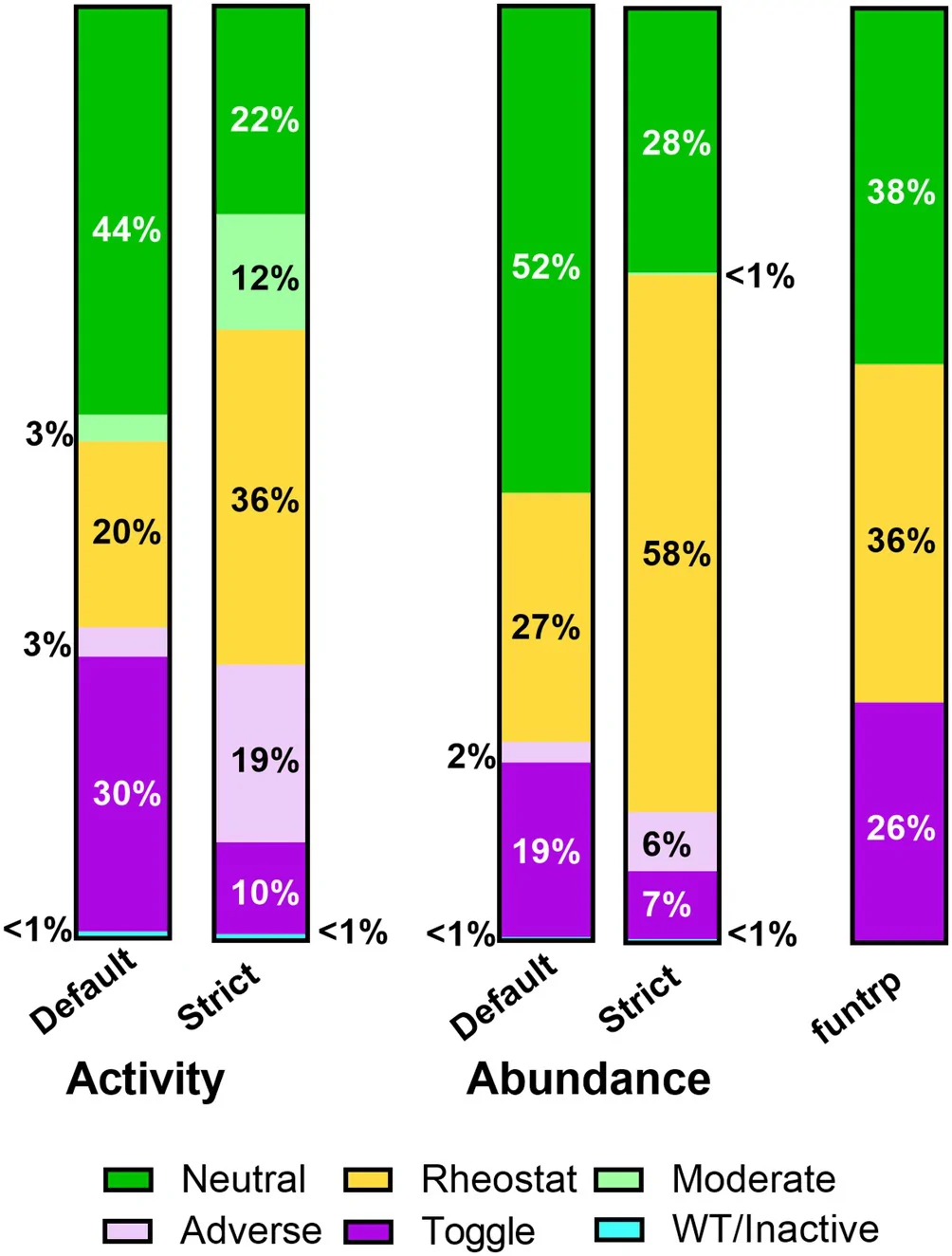
Comparison of PLpro experimental and predicted position classes. The percent of positions in the PLpro activity and abundance datasets that were experimentally assigned to each class were determined using (i) RheoScale 2.0 default or (ii) strict toggle/neutral thresholds. For the activity data, 30 positions changed from neutral to moderate, 50 changed from toggle to adverse, 38 changed from neutral to rheostat, and 12 changed from toggle to rheostat; 101 neutral and toggle position assignments stayed the same. For the abundance data, 1 position changed from neutral to moderate, 13 changed from toggle to adverse, 74 changed from neutral to rheostat, and 23 changed from toggle to rheostat; 112 neutral and toggle assignments stayed the same. The greater number of rheostat re‐assignments in the abundance data is because RheoScale analyses used fewer histogram bins (7) than the activity data (10); this was due to the lower experimental resolution of the abundance data. The right‐most bar shows the predicted distribution of positions among the three funtrp classes (neutral, rheostat, and toggle).

Our second question was to examine the effects of stricter thresholds. Results showed that the number of positions that were assigned as neutral or toggle decreased in both datasets, as expected (Figure 6). Many of the default‐neutral and ‐toggle positions were reassigned to the moderate and adverse classes, respectively, because variants that fell outside of the neutral/toggle bins were located in nearby bins. This is consistent with the emerging picture that individual positions fall along a continuum of substitution sensitivities. Other positions changed from neutral (or toggle) to rheostat assignments. This arose when the remaining variants widely sampled the rest of the histogram bins; that is, positions with five non‐neutral (or non‐toggle) variants could occupy enough bins to reach a rheostat score ≥0.5.

Our third question was related to the overall success of funtrp for the two datasets. For these comparisons, the experimentally‐moderate and ‐adverse assignments were re‐classified as rheostat positions. Using the RheoScale default thresholds, funtrp's overall prediction success was greater than 50% for both experimental datasets, which exceeds random chance (33% for a three‐category assignment). We also considered that the mismatch could arise from either a false prediction or an experimental misclassification. However, there was no correlation between prediction accuracy and the number of variants available to make the experimental assignment, reducing the likelihood that a false prediction arises from a false experimental assignment. Instead, prediction accuracy differed among position classes, with experimentally‐neutral and ‐toggle positions better predicted than rheostat positions (Table 1).

**TABLE 1:** Statistical analyses of funtrp predictions for PLpro position classes.

	Correct predictions	TC^a^ (N)	TC (R)	TC (T)	FC^b^ (T|N)	FC (R|N)	FC (T|R)	FC (N|R)	FC (R|T)	FC (N|T)
Experimental assignments used 10 bins, default score thresholds^c^										
Activity										
Number	181	90	40	51	8	40	21	21	33	9
Fraction	0.57	0.65	0.49	0.55	0.06	0.29	0.26	0.26	0.35	0.10
Abundance										
Number	167	97	40	30	15	52	36	15	21	8
Fraction	0.53	0.59	0.44	0.51	0.09	0.32	0.40	0.16	0.36	0.14
Experimental assignments used 10 bins, strict score thresholds^d^										
Activity										
Number	163	50	90	23	2	18	56	68	6	2
Fraction	0.52	0.71	0.42	0.74	0.03	0.26	0.26	0.32	0.19	0.06
Abundance										
Number	152	57	80	15	5	27	61	62	7	1
Fraction	0.48	0.64	0.39	0.65	0.06	0.30	0.30	0.31	0.30	0.04

^a^ “TC(*i*)”: Given the experimental “truth” for class *i*, how many positions did funtrp predict correctly?

^b^ “FC(*i*|*j*)”: Given the experimental “truth” for class *j*, how many positions were misclassified as *i*?

^c^ Rheoscale default score thresholds: Neutral score 0.7, Toggle score 0.64, Rheostat score 0.5.

^d^ Strict score thresholds: Neutral score 0.9, Toggle score 0.9, Rheostat score 0.5.

When we used stricter score thresholds for assigning experimentally‐neutral and ‐toggle positions, funtrp prediction accuracy increased for these two classes (Table 1). However, the accuracy for predicted rheostat positions decreased (Table 1), as did the overall accuracy. This could be because the toggle and neutral positions with the most extreme substitution sensitivities also have the most extreme conserved and non‐conserved ConSurf scores, respectively (Martin et al., 2020; Swint‐Kruse et al., 2021). Given the importance of ConSurf scores to funtrp predictions, the funtrp toggle positions may be among the most conserved PLpro positions; likewise, the funtrp neutral positions may be among the least conserved.

Thus, positions with intermediate conservation remain among the hardest to predict for both position classes and substitution outcomes. We hypothesize that the discrepancies might be explained by our previous findings in a range of proteins, where outcomes at rheostat positions were not well‐explained by the common structural properties (Swint‐Kruse & Fenton, 2024) that are used as other input in funtrp. One possible route for improving predictions is the addition of more protein features; for example, we found that information about coupled dynamic motions was able to discriminate neutral and rheostat positions in the lactose repressor protein (Campitelli et al., 2024). Alternatively, the multiple sequence alignment created by ConSurf could be improved for PLpro via manual curation (which is not currently an option in funtrp); in our hands, we have found that the over‐representation of SARS‐CoV2 sequences makes it difficult for automated searches to identify the broadest possible range of homologs.

Finally, we note that all activity classes were better predicted than abundance classes (Table 1). This is consistent with the fact that both the activity measurements and the evolutionary information used in funtrp/ConSurf are sensitive to both changes in catalytic activity and abundance. As such, funtrp—which is trained on fitness data that likely contains information from variant effects on both function and abundance—might be more suitable for predicting the composite sensitivity of each position rather than position roles in specific biochemical features, such as binding affinities, allosteric response, or stability.

## 4 CONCLUSION

By capturing both the extreme and intermediate substitution outcomes, RheoScale 2.0 reveals position‐specific behaviors that are obscured by average values (Figure 5). Understanding these intermediate outcomes, and the rheostat positions that host such changes, can help prioritize research for mechanistic studies, protein engineering, and drug development. For example, the enrichment of inhibitor‐resistance substitutions at rheostat positions in PLpro suggests that substitutions at rheostat positions provide accessible routes for viral evolution and drug escape.

The current results demonstrate that RheoScale 2.0 assignments (i) are robust to variations in subjective analysis parameters and (ii) that position assignments are >90% accurate with as few as 13 variants per position, which can ameliorate the costs and time required to generate all 19 variants for every position. The experimental error, the dynamic range of the assay, and data resolution remain important considerations for revealing the evolutionarily‐important intermediate outcomes at rheostat, moderate, and adverse positions.

The PLpro example that we used to carry out these statistical assessments of RheoScale 2.0 analyses also illustrates an important characteristic of the protein sequence‐structure–function relationship: A given amino acid position can have more than one role in the protein architecture, and the position can have different substitution sensitivities for its different roles (i.e., catalytic activity, abundance/stability, or inhibitor response). Thus, it is imperative that researchers recognize the feature(s) detected by each assay and understand when or if some substitution effects are concealed by assay design.

In summary, RheoScale 2.0 provides an objective framework for extracting position specific information from high‐throughput datasets in both Excel and Python formats. Applying the framework across multiple assays and proteins will deepen our understanding of sequence‐structure–function relationships, thereby improving our abilities to interpret pathological variants and identify positions well‐suited for protein engineering and drug development.

## AUTHOR CONTRIBUTIONS

**Shwetha Sreenivasan:** Writing – original draft; writing – review and editing; methodology; software; formal analysis; validation; visualization; investigation; conceptualization. **Carter J. Gray:** Methodology; writing – review and editing; formal analysis; software; validation; visualization; investigation; conceptualization. **Liskin Swint‐Kruse:** Writing – original draft; writing – review and editing; formal analysis; project administration; supervision; conceptualization; validation; methodology; visualization; investigation; data curation; funding acquisition; resources. **Hannah C. Cleveland:** Writing – review and editing; methodology; software; validation; investigation. **David H. Liu:** Writing – original draft; writing – review and editing; methodology; formal analysis; software; validation; visualization; investigation; conceptualization.

## CONFLICT OF INTEREST STATEMENT

The authors declare that they have no known competing financial interests or personal relationships that could have appeared to influence the work reported in this paper.

## Data Availability

The data that support the findings of this study are openly available in GitHub at https://github.com/liskinsk/RheoScale-calculator.

## References

[pro70804-bib-0001] Amorosi CJ , Chiasson MA , McDonald MG , Wong LH , Sitko KA , Boyle G , et al. Massively parallel characterization of CYP2C9 variant enzyme activity and abundance. Am J Hum Genet. 2021;108:1735–1751. 10.1016/j.ajhg.2021.07.001 PMC8456167 34314704

[pro70804-bib-0002] Ashkenazy H , Abadi S , Martz E , Chay O , Mayrose I , Pupko T , et al. ConSurf 2016: an improved methodology to estimate and visualize evolutionary conservation in macromolecules. Nucleic Acids Res. 2016;44:W344–W350. 10.1093/nar/gkw408 PMC4987940 27166375

[pro70804-bib-0003] Ashkenazy H , Erez E , Martz E , Pupko T , Ben‐Tal N . ConSurf 2010: calculating evolutionary conservation in sequence and structure of proteins and nucleic acids. Nucleic Acids Res. 2010;38:W529–W533. 10.1093/nar/gkq399 PMC2896094 20478830

[pro70804-bib-0004] Campitelli P , Ross D , Swint‐Kruse L , Ozkan SB . Dynamics‐based protein network features accurately discriminate neutral and rheostat positions. Biophys J. 2024;123:3612–3626. 10.1016/j.bpj.2024.09.013 PMC11494493 39277794

[pro70804-bib-0005] Campitelli P , Swint‐Kruse L , Ozkan S . Substitutions at non‐conserved rheostat positions modulate function by Re‐wiring long‐range, dynamic interactions. Mol Biol Evol. 2021;38:201–214. 10.1093/molbev/msaa202 PMC7783170 32780837

[pro70804-bib-0006] Fenton KD , Meneely KM , Wu T , Martin TA , Swint‐Kruse L , Fenton AW , et al. Substitutions at a rheostat position in human aldolase a cause a shift in the conformational population. Protein Sci. 2022;31:357–370. 10.1002/pro.4222 PMC8819835 34734672

[pro70804-bib-0007] Flynn JM , Samant N , Schneider‐Nachum G , Barkan DT , Yilmaz NK , Schiffer CA , et al. Comprehensive fitness landscape of SARS‐CoV‐2 Mpro reveals insights into viral resistance mechanisms. Elife. 2022;11:e77433. 10.7554/eLife.77433 PMC9323007 35723575

[pro70804-bib-0008] Flynn JM , Zvornicanin SN , Tsepal T , Shaqra AM , Kurt Yilmaz N , Jia W , et al. Contributions of hyperactive mutations in M(pro) from SARS‐CoV‐2 to drug resistance. ACS Infect Dis. 2024;10:1174–1184. 10.1021/acsinfecdis.3c00560 PMC11179160 38472113

[pro70804-bib-0009] Gray CJ , O'Neil PT , Schwingen KM , Hillebrand C , Swint‐Kruse L . Expanding the toolkit of LacI/GalR chimeras. PLoS One. 2026;21:e0345158. 10.1371/journal.pone.0345158 PMC13056197 41945592

[pro70804-bib-0010] Harris CR , Millman KJ , van der Walt SJ , Gommers R , Virtanen P , Cournapeau D , et al. Array programming with NumPy. Nature. 2020;585:357–362. 10.1038/s41586-020-2649-2 PMC7759461 32939066

[pro70804-bib-0011] Hodges AM , Fenton AW , Dougherty LL , Overholt AC , Swint‐Kruse L . RheoScale: a tool to aggregate and quantify experimentally determined substitution outcomes for multiple variants at individual protein positions. Hum Mutat. 2018;39:1814–1826. 10.1002/humu.23616 PMC6602090 30117637

[pro70804-bib-0012] Hunter JD . Matplotlib: a 2D graphics environment. Comput Sci Eng. 2007;9:90–95.

[pro70804-bib-0013] Kariyawasam NL , Sivchenko A , Swint‐Kruse L , Smith PE . Substitutions at rheostat position 52 of LacI have long‐range effects on the LacI conformational landscape. Biophys Chem. 2025;320‐321:107414. 10.1016/j.bpc.2025.107414 PMC11893255 39987706

[pro70804-bib-0014] Klemm T , Ebert G , Calleja DJ , Allison CC , Richardson LW , Bernardini JP , et al. Mechanism and inhibition of the papain‐like protease, PLpro, of SARS‐CoV‐2. EMBO J. 2020;39:e106275. 10.15252/embj.2020106275 PMC7461020 32845033

[pro70804-bib-0015] Livesey BJ , Marsh JA . Variant effect predictor correlation with functional assays is reflective of clinical classification performance. Genome Biol. 2025;26:104. 10.1186/s13059-025-03575-w PMC12016141 40264194

[pro70804-bib-0016] López SG , Salomon J , Boomsma W . Supervised learning of protein melting temperature: cross‐species vs. species‐specific prediction. Proteins Struct Funct Bioinform. 2025;93:2158–2166. 10.1002/prot.70019 PMC12594180 40653882

[pro70804-bib-0017] Markiewicz P , Kleina LG , Cruz C , Ehret S , Miller JH . Genetic studies of the lac repressor. XIV. Analysis of 4000 altered Escherichia coli lac repressors reveals essential and non‐essential residues, as well as “spacers” which do not require a specific sequence. J Mol Biol. 1994;240:421–433. 10.1006/jmbi.1994.1458 8046748

[pro70804-bib-0018] Martin TA , Wu T , Tang Q , Dougherty LL , Parente DJ , Swint‐Kruse L , et al. Identification of biochemically neutral positions in liver pyruvate kinase. Proteins Struct Funct Bioinform. 2020;88:1340–1350. 10.1002/prot.25953 PMC8990530 32449829

[pro70804-bib-0019] McKinney W . Data structures for statistical computing in python. Scipy. 2010;445:51–56.

[pro70804-bib-0020] Meinhardt S , Manley MW Jr , Parente DJ , Swint‐Kruse L . Rheostats and toggle switches for modulating protein function. PLoS One. 2013;8:e83502. 10.1371/journal.pone.0083502 PMC3875437 24386217

[pro70804-bib-0021] Miller M , Bromberg Y , Swint‐Kruse L . Computational predictors fail to identify amino acid substitution effects at rheostat positions. Sci Rep. 2017;7:41329. 10.1038/srep41329 PMC5278360 28134345

[pro70804-bib-0022] Miller M , Vitale D , Kahn PC , Rost B , Bromberg Y . Funtrp: identifying protein positions for variation driven functional tuning. Nucleic Acids Res. 2019;47:e142. 10.1093/nar/gkz818 PMC6868392 31584091

[pro70804-bib-0023] O'Neil PT , Swint‐Kruse L , Fenton AW . Rheostatic contributions to protein stability can obscure a position's functional role. Protein Sci. 2024;33:e5075. 10.1002/pro.5075 PMC11187868 38895978

[pro70804-bib-0024] Page BM , Martin TA , Wright CL , Fenton LA , Villar MT , Tang Q , et al. Odd one out? Functional tuning of *Zymomonas mobilis* pyruvate kinase is narrower than its allosteric, human counterpart. Protein Sci. 2022;31:e4336. 10.1002/pro.4336 PMC9202079 35762709

[pro70804-bib-0025] Pettersen EF , Goddard TD , Huang CC , Meng EC , Couch GS , Croll TI , et al. UCSF ChimeraX: structure visualization for researchers, educators, and developers. Protein Sci. 2021;30:70–82. 10.1002/pro.3943 PMC7737788 32881101

[pro70804-bib-0026] Ruggiero MJ , Malhotra S , Fenton AW , Swint‐Kruse L , Karanicolas J , Hagenbuch B . A clinically‐relevant polymorphism in the Na(+)/taurocholate cotransporting polypeptide (NTCP) occurs at a rheostat position. J Biol Chem. 2021;296:100047. 10.1074/jbc.RA120.014889 PMC7948949 33168628

[pro70804-bib-0027] Sreenivasan S , Fontes JD , Swint‐Kruse L . Dissecting the effects of single amino acid substitutions in SARS‐CoV‐2 Mpro. Protein Sci. 2025;34:e70225. 10.1002/pro.70225 PMC12267653 40671356

[pro70804-bib-0028] Sreenivasan S , Heffren P , Suh KS , Rodnin MV , Kosa E , Fenton AW , et al. The intrinsically disordered transcriptional activation domain of CIITA is functionally tuneable by single substitutions: an exception or a new paradigm? Protein Sci. 2024;33:e4863. 10.1002/pro.4863 PMC10806935 38073129

[pro70804-bib-0029] Swint‐Kruse L . Using evolution to guide protein engineering: the devil IS in the details. Biophys J. 2016;111:10–18. 10.1016/j.bpj.2016.05.030 PMC4945580 27410729

[pro70804-bib-0030] Swint‐Kruse L , Dougherty LL , Page B , Wu T , O'Neil PT , Prasannan CB , et al. PYK‐SubstitutionOME: an integrated database containing allosteric coupling, ligand affinity and mutational, structural, pathological, bioinformatic and computational information about pyruvate kinase isozymes. Database. 2023;2023:baad030. 10.1093/database/baad030 PMC10176505 37171062

[pro70804-bib-0031] Swint‐Kruse L , Fenton AW . Rheostats, toggles, and neutrals, oh my! A new framework for understanding how amino acid changes modulate protein function. J Biol Chem. 2024;300:105736. 10.1016/j.jbc.2024.105736 PMC10914490 38336297

[pro70804-bib-0032] Swint‐Kruse L , Martin TA , Page BM , Wu T , Gerhart PM , Dougherty LL , et al. Rheostat functional outcomes occur when substitutions are introduced at nonconserved positions that diverge with speciation. Protein Sci. 2021;30:1833–1853. 10.1002/pro.4136 PMC8376419 34076313

[pro70804-bib-0033] Swint‐Kruse L , Ruggiero MJ , Hagenbuch B . Amino acid substitutions at rheostat positions in the Na+/taurocholate cotransporting polypeptide substrate channel have pleiotropic effects. Protein Sci. 2025;34:e70313. 10.1002/pro.70313 PMC12521614 41088770

[pro70804-bib-0034] Tack DS , Tonner PD , Pressman A , Olson ND , Levy SF , Romantseva EF , et al. The genotype‐phenotype landscape of an allosteric protein. Mol Syst Biol. 2021;17:e10179. 10.15252/msb.202010179 PMC8009258 33784029

[pro70804-bib-0035] Tang Q , Fenton AW . Whole‐protein alanine‐scanning mutagenesis of allostery: a large percentage of a protein can contribute to mechanism. Hum Mutat. 2017;38:1132–1143. 10.1002/humu.23231 PMC5561450 28407397

[pro70804-bib-0036] Turina P , Petrosino M , Enriquez Sandoval CA , Novak L , Pasquo A , Alexov E , et al. Assessing the predicted impact of single amino acid substitutions in MAPK proteins for CAGI6 challenges. Hum Genet. 2025;144:265–280. 10.1007/s00439-024-02724-8 PMC11975483 39976676

[pro70804-bib-0037] Wu T , Swint‐Kruse L , Fenton AW . Functional tunability from a distance: rheostat positions influence allosteric coupling between two distant binding sites. Sci Rep. 2019;9:16957. 10.1038/s41598-019-53464-z PMC6861286 31740686

[pro70804-bib-0038] Wu X , Devine SM , Go M , Nguyen JV , Lu BGC , Loi K , et al. Divergent resistance pathways amongst SARS‐CoV‐2 PLpro inhibitors highlight the need for scaffold diversity. PLoS Pathog. 2025;21:e1013468. 10.1371/journal.ppat.1013468 PMC12431669 40911642

[pro70804-bib-0039] Wu X , Go M , Nguyen JV , Kuchel NW , Lu BGC , Zeglinski K , et al. Mutational profiling of SARS‐CoV‐2 papain‐like protease reveals requirements for function, structure, and drug escape. Nat Commun. 2024;15:6219. 10.1038/s41467-024-50566-9 PMC11266423 39043718

[pro70804-bib-0040] Zhang H , Kabir M , Ahmed S , Vihinen M . There will always be variants of uncertain significance. Analysis of VUSs. NAR Genom Bioinform. 2024;6:lqae154. 10.1093/nargab/lqae154 PMC11616676 39633727

